# Supplementary material for: Non-invasive measurement of mRNA decay reveals translation initiation as the major determinant of mRNA stability
Source: eLife. 2018 Sep 7;7:e32536. doi: 10.7554/eLife.32536 (PMC6152797; doi:10.7554/eLife.32536)
Supplement: Supplementary file 1. [file elife-32536-supp1.docx]

Supplemental File 1

parameter reference

| ORF length and GC content | calculated from W303 genome files (SGD W303_ALAV00000000 2012 assembly) |
| --- | --- |
| abundance and UTR lengths | “The transcriptional landscape of the yeast genome defined by RNA sequencing.” Nagalakshmi U, Wang Z, Waern K, Shou C, Raha D, Gerstein M, Snyder M. Science. 2008 Jun 6;320(5881):1344-9. |
| polyA length | “Poly(A)-tail profiling reveals an embryonic switch in translational control.” Subtelny AO, Eichhorn SW, Chen GR, Sive H, Bartel DP. Nature. 2014 Apr 3;508(7494):66-71. |
| translational efficiency | “High-resolution view of the yeast meiotic program revealed by ribosome profiling.” Brar GA1, Yassour M, Friedman N, Regev A, Ingolia NT, Weissman JS. Science. 2012 Feb 3;335(6068):552-7. |
| CAI | “A single determinant dominates the rate of yeast protein evolution.” Drummond DA, Raval A, Wilke CO. Mol Biol Evol. 2006 Feb;23(2):327-37. |
| nTE | “Evolutionary conservation of codon optimality reveals hidden signatures of cotranslational folding.” Pechmann S, Frydman J. Nat Struct Mol Biol. 2013 Feb;20(2):237-43. |
