# Supplementary material for: Non-invasive measurement of mRNA decay reveals translation initiation as the major determinant of mRNA stability
Source: eLife. 2018 Sep 7;7:e32536. doi: 10.7554/eLife.32536 (PMC6152797; doi:10.7554/eLife.32536)
Supplement: Supplementary file 2. [file elife-32536-supp2.docx]

# Supplemental File 2

# strain genotype

KWY165 MATa leu2-3,112 trp1-1 can1-100 ura3-1 ade2-1 his3-11,15 [phi+]

KWY1601 *MATa his3Δ1 leu2Δ0 met15Δ0 ura3Δ0*

KWY3238 MATa leu2-3,112 trp1-1 can1-100 ura3-1 ade2-1 his3-11,15 [phi+] dhh1∆::KanMX6 Dcp2-RFP::NatMX6 pRS316-pDHH1-DHH1-GFP

KWY5242 MATa leu2-3,112 trp1-1 can1-100 ura3-1 ade2-1 his3-11,15 [phi+] dhh1∆::KanMX6 DCP2-mCherry::NatMX6 pRS316-pDHH1- DHH1(R322A,S340A,R370A)-GFP

KWY5244 MATa leu2-3,112 trp1-1 can1-100 ura3-1 ade2-1 his3-11,15 [phi+] dhh1∆::KanMX6 DCP2-mCherry::NatMX6 pRS316-pDHH1-DHH1(F66R, Q73A)-GFP

KWY5246 MATa leu2-3,112 trp1-1 can1-100 ura3-1 ade2-1 his3-11,15 [phi+] dhh1∆::KanMX6 DCP2-mCherry::NatMX6 pRS316-pDHH1-GFP

KWY5948 MATa leu2-3,112 trp1-1 can1-100 ura3-1 ade2-1 his3-11,15 [phi+] DHH1-yEGFP::CaURA3MX6 DCP2-mCherry::NatMX6

KWY6554 MATa leu2-3,112 trp1-1 can1-100 ura3-1 ade2-1 his3-11,15 [phi+] PAB1- GFP::HisMX6 DCP2-mCherry-NatMX6

KWY6963 MATa leu2-3,112 trp1-1 can1-100 ura3-1 ade2-1 his3-11,15 [phi+] DCP2- GFP::HisMX6 PGK1-24xPP7sl YCplac33-pMET25-PP7CP-mKate2

KWY7227 MATa met15∆0 ura3∆0 his3∆1∆::CG-HIS3::pGPD1-LexA-B112 leu2∆0::pMET25-PP7CP-yEGFP::LEU2 Nup60-3xmKate2::CaURA3

SCO2::p4xLexOcyc1-3xGST-V5-24xPP7sl-tCYC1-NatNT2 Dcp2- mCherry::KanMX6

KWY7245 MATa leu2-3,112 trp1-1 can1-100 ura3-1 ade2-1 his3-11,15 [phi+] DCP2- GFP::HisMX6 FBA1-24xPP7sl YCplac33-pMET25-PP7CP-mKate2

KWY7246 MATa his3Δ1 leu2Δ0 met15Δ0 ura3Δ0 GFA1-24xPP7sl Dcp2- GFP::HisMX6 YCplac33-pMET25-PP7CP-mKate2

KWY7324 MATa leu2-3,112 trp1-1 can1-100 ura3-1 ade2-1 his3-11,15 [phi+] pRS425

KWY7325 MATa leu2-3,112 trp1-1 can1-100 ura3-1 ade2-1 his3-11,15 [phi+] CDC33-IAA7-3V5::KanMx6

KWY7326 MATa trp1-1 can1-100 ura3-1 ade2-1 his3-11,15 [phi+] leu-2- 3,112∆::CG-LEU2::pGPD1-OsTIR1 CDC33-IAA7-3V5::KanMx6

KWY7327 MATa leu2-3,112 can1-100 ura3-1 ade2-1 his3-11,15 [phi+] trp1-1∆::CG- TRP1::pGPD1-LexA-B112 pRS425

KWY7328 MATa leu2-3,112 can1-100 ura3-1 ade2-1 his3-11,15 [phi+] trp1-1∆::CG- TRP1::pGPD1-LexA-B112 pRS425-p4LexOCYC1-CDC33-3V5

KWY7329 MATa leu2-3,112 can1-100 ura3-1 ade2-1 his3-11,15 [phi+] trp1-1∆::CG- TRP1::pGPD1-LexA-B112 pRS425-p4LexOCYC1-CDC33

KWY7330 MATa leu2-3,112 can1-100 ura3-1 ade2-1 his3-11,15 [phi+] trp1-1∆::CG- TRP1::pGPD1-LexA-B112 pRS425-p4LexOCYC1-cdc33ΔG

KWY7331 MATa leu2-3,112 can1-100 ura3-1 ade2-1 his3-11,15 [phi+] trp1-1∆::CG- TRP1::pGPD1-LexA-B112 pRS425-p4LexOCYC1-cdc33∆CAP

KWY7332 MATa leu2-3,112 can1-100 ura3-1 ade2-1 his3-11,15 [phi+] trp1-1∆::CG- TRP1::pGPD1-LexA-B112 pRS425-p4LexOCYC1-cdc33∆G∆CAP

KWY7333 MATa trp1-1 can1-100 ura3-1 ade2-1 his3-11,15 [phi+] leu-2- 3,112∆::CG-LEU2::pGPD1-OsTIR1

KWY7334 MATa leu2-3,112 can1-100 ura3-1 ade2-1 [phi+] trp1-1∆::CG- TRP1::pGPD1-LexA-B112 his3-11,15∆::CG-HIS3::pGPD1-OsTIR1 CDC33- IAA7-3V5::KanMx6 pRS425-p4LexOCYC1-cdc33∆CAP

KWY7335 MATa leu2-3,112 trp1-1 can1-100 ura3-1 ade2-1 his3-11,15 [phi+] RPL28(Q38K)

KWY7336 MATa leu2-3,112 can1-100 ura3-1 ade2-1 his3-11,15 [phi+] trp1-1∆::CG- TRP1::pGPD1-LexA-B112 CDC33-IAA7-3V5::KanMx6 pRS425

KWY7337 MATa leu2-3,112 trp1-1 can1-100 ura3-1 ade2-1 [phi+] HIS3 gcn2∆::KanMx6

KWY7338 MATa leu2-3,112 can1-100 ura3-1 ade2-1 his3-11,15 [phi+] trp1-1∆::CG- TRP1::pGPD1-LexA-B112 pRS425-p4xLexOcyc1-cdc33∆G-3V5

KWY7339 MATa leu2-3,112 can1-100 ura3-1 ade2-1 his3-11,15 [phi+] trp1-1∆::CG- TRP1::pGPD1-LexA-B112 pRS425-p4xLexOcyc1-cdc33∆CAP-3V5

KWY7340 MATa leu2-3,112 can1-100 ura3-1 ade2-1 his3-11,15 [phi+] trp1-1∆::CG- TRP1::pGPD1-LexA-B112 pRS425-p4xLexOcyc1-cdc33∆G∆CAP-3V5

KWY7341 MATa leu2-3,112 trp1-1 can1-100 ura3-1 ade2-1 [phi+] his3-11,15∆::CG- HIS3::pGPD1-OsTIR1 CDC33-IAA7-3V5::KanMx6 pRS425
