# Supplementary material for: Non-invasive measurement of mRNA decay reveals translation initiation as the major determinant of mRNA stability
Source: eLife. 2018 Sep 7;7:e32536. doi: 10.7554/eLife.32536 (PMC6152797; doi:10.7554/eLife.32536)
Supplement: Supplementary file 3. [file elife-32536-supp3.docx]

Supplemental File 3

pKW1643 pAS439-*rcc1* (Xl) pKW1644 pAS439-*srp1α* (Hs) pKW2306 pRS316-*pDHH1-GFP* pKW2312 pRS316-*pDHH1-DHH1-GFP*

pKW2550 pFA6a-*24xPP7sl-tCYC1-KanMX6* pKW2830 pNH605-*pGPD1-OsTIR1*

pKW2867 pRS316-*pDHH1-DHH1(R322A,S340A,R370A)-GFP* pKW2874 pNH603-*pGPD1-OsTIR1*

pKW3072 pRS316-*pDHH1-DHH1(F66R, Q73A)-GFP* pKW3616 pRS305-*pMET25-PP7CP-yEGFP*

pKW3800 YCplac33-*pMET25-PP7CP-mKate2* pKW3908 pNH604-*pGPD1-LexA-B112*

pKW4019 pFA6a-*3xmKate2-CaURA3MX6* pKW4073 pNH603-*pGPD1-LexA-B112*

pKW4190 pRS313-*HR1_Chr2(SCO2)-p4xLexOcyc1-3xGST-V5-24xPP7sl-tCYC1-NatNT2-*

*HR2_Chr2(SCO2)* pKW4325 pFA6a-*IAA7-3V5-KanMX6*

pKW4326 pRS425-*p4xLexOcyc1-CDC33-3V5*

pKW4327 pRS425-*p4xLexOcyc1-CDC33* pKW4328 pRS425-*p4xLexOcyc1-cdc33∆G* pKW4329 pRS425-*p4xLexOcyc1-cdc33∆cap* pKW4330 pRS425-*p4xLexOcyc1-cdc33∆G∆cap* pKW4331 pRS425-*p4xLexOcyc1-cdc33∆G-3V5* pKW4332 pRS425-*p4xLexOcyc1-cdc33∆cap-3V5* pKW4333 pRS425-*p4xLexOcyc1-cdc33∆G∆cap-3V5*
