# Supplementary material for: Non-invasive measurement of mRNA decay reveals translation initiation as the major determinant of mRNA stability
Source: eLife. 2018 Sep 7;7:e32536. doi: 10.7554/eLife.32536 (PMC6152797; doi:10.7554/eLife.32536)
Supplement: Supplementary file 4. [file elife-32536-supp4.docx]

# Supplemental File 4

# target forward reverse

*ACT1* tggattccggtgatggtgtt tcaaaatggcgtgaggtagaga *AIM13* ggcaaaattgactgagtgtc acccataacgagcttcttg *AIM7* gcattagaaatgatcagagagg cctcaattcttcaacgtctg *CIS3* accttgaaggacggtgttttg tggtggaccgtcaaattgg *CYS4* ggaaaccgctaaggtcactg tgccgtcttcagtcaacacag *DED1* agcaacaaccgtcgtggtgg gaacggccgttagatctgctgc *srp1α* (Hs) gtggaaaatcagctccaagc aaccagcccggattatgttg *NPA3* tgatggtcttgtggataggg agagttctcgttgacctcac *OSW5* cttctaaattcgtcaccgaag tgagagctggaacttattacc *PAM16* gagcaaagtttatcgagcagc gcttgcactattatctgcacc *PDC1* gcttgaagccatacttgttcg ttgagcctttggaccgtg *RPC11* tggtggtgagagtgcttac tccttccatctatgaccacag *RPL25* tatgcttccaaggctgttcc gcggtttcagaagtgattgg *RPS2* accttgaaggctgctttcg aaactggcaatggttgttcg *TPI1* gctgctactccagaagatgctc cagccttgtcacccaacttg *VMA21* cagcaatttacgccaaatacc gaacgctacaacaatgtaaacg *rcc1* (Xl) gcttatagtcttggccgtgc taagtcaggaattggggtgg
